# Carnosine protects human microglia against Aβ oligomers through a multimodal mechanism of action: inhibition of oxidative stress, rescue of cellular energy status, and enhancement of phagocytosis

**DOI:** 10.3389/fimmu.2026.1768094

**Published:** 2026-02-13

**Authors:** Anna Privitera, Vincenzo Cardaci, Matthew C. Zupan, Lucia Di Pietro, Giuseppe Carota, Jay Sibbitts, Renata Mangione, Andrea Graziani, Lucia Buccarello, Francesco Bellia, Valentina Di Pietro, Giuseppe Lazzarino, Susan M. Lunte, Meredith D. Hartley, Filippo Caraci, Barbara Tavazzi, Emiliano Maiani, Angela M. Amorini, Giacomo Lazzarino, Giuseppe Caruso

**Affiliations:** 1 Department of Drug and Health Sciences, University of Catania, Catania, Italy; 2 Department of Biomedical and Biotechnological Sciences, University of Catania, Catania, Italy; 3 CRA Mirabilis, Fondazione Mantovani, Milan, Arconate (MI), Italy; 4 Department of Chemistry, University of Kansas, Lawrence, KS, United States; 5 Scuola Superiore di Catania, University of Catania, Catania, Italy; 6 Ralph N. Adams Institute for Bioanalytical Chemistry, University of Kansas, Lawrence, KS, United States; 7 Departmental Faculty of Medicine, UniCamillus—Saint Camillus International University of Health and Medical Sciences, Rome, Italy; 8 IRCCS San Camillo Hospital, Venice, Italy; 9 Department of Inflammation and Ageing, College of Medicine and Health, University of Birmingham, Birmingham, United Kingdom; 10 LTA Biotech S.r.l., Catania, Italy; 11 Department of Pharmaceutical Chemistry, University of Kansas, Lawrence, KS, United States; 12 Unit of Neuropharmacology and Translational Neurosciences, Oasi Research Institute-IRCCS, Troina, Italy

**Keywords:** Alzheimer’s disease, carnosine, energy metabolism, human microglia, neurodegeneration, oxidative stress

## Abstract

**Introduction:**

Carnosine is an endogenous dipeptide composed by β-alanine and L-histidine widely distributed in excitable tissues like muscles and brain. Carnosine participates in the cellular defenses against oxidative/nitrosative stress through a multimodal mechanism of action, including scavenging of the reactive oxygen and nitrogen species (ROS and RNS) and, in brain cells, the inhibition of amyloid-beta (Aβ) aggregation. Microglia play a central role in the pathophysiology of Alzheimer’s disease (AD), maintaining the homeostasis of the brain microenvironment. However, its hyperactivation causes an increased secretion of inflammatory mediators and free radicals, leading to neuroinflammatory phenomena that exacerbate neurodegeneration. In the present work, carnosine was tested for its ability to protect human microglial cells (HMC3) against Aβ oligomers-induced oxidative stress and energy metabolism unbalance.

**Methods:**

The effects of carnosine to modulate nitric oxide (NO) and ROS intracellular levels were evaluated by microchip electrophoresis coupled to laser-induced fluorescence (ME-LIF), while additional stress-related parameters and cellular energy metabolism were investigated through high-performance liquid chromatography (HPLC).

**Results:**

Pre-treatment with carnosine counteracted the oxidative/nitrosative stress induced by Aβ1-42 oligomers by decreasing the intracellular levels of NO and ROS, and rescuing GSH levels. Carnosine preserved cellular mitochondrial-related energy metabolism, restoring concentrations of high-energy phosphates, nicotinic coenzymes and oxypurines, and normalizing UDP-derivatives homeostasis. Furthermore, carnosine strongly enhanced the phagocytic activity of HMC3 cells.

**Discussion/Conclusion:**

These results demonstrate the protective effects of carnosine on human microglial cells against detrimental alterations induced by Aβ oligomers, underlining the multimodal mechanism of action of this dipeptide and supporting its promising potential in the context of AD pathology.

## 1 Introduction

Carnosine, a naturally occurring dipeptide composed of beta-alanine and L-histidine, is also available as an over-the-counter dietary supplement (1). It is distributed in various mammalian tissues, with particularly high concentrations found in the brain and in skeletal and cardiac muscles (2, 3).

Pre-clinical studies have demonstrated that carnosine has significant neuroprotective and anti-inflammatory properties (4). These effects are exerted through multiple mechanisms of action that include the scavenging of free radicals and the inhibition of toxic protein aggregation (5, 6). Additionally, carnosine is also able to decrease the production of pro-inflammatory mediators (7), to modulate the response of immune cells such as macrophages and microglia, as well as to regulate the production of reactive oxygen (ROS) and nitrogen (RNS) species (8, 9). Two very recent *in vivo* studies carried by some of us have also shown the pro-cognitive effects exerted by this dipeptide, with carnosine mitigating cognitive impairment and dopamine release in an okadaic acid-induced zebrafish model with Alzheimer’s disease (AD)-like symptoms (10) or reverting the memory aversive states induced by neuroinflammation in *Lymnaea stagnalis* (11). Interestingly, clinical studies suggested the potential benefits of carnosine in preserving mental health and function in aging populations enhancing the overall cognitive function (12, 13). Carnosine therapeutic potential has been explored in various neurodegenerative and neuropsychiatric disorders, including Parkinson’s disease (PD), schizophrenia, AD, attention-deficit/hyperactivity disorder, and age-related cognitive decline (12, 14–17).

Microglia are the immune cells of the central nervous system (CNS) and are crucial for brain development, memory, synaptic plasticity, and neurogenesis (18). Microglial cells can be divided into differently functional populations, with M1 (classically activated or pro-inflammatory) and M2 (alternatively activated or anti-inflammatory) being the most notable. The M1 phenotype is prone to increase the production of pro-inflammatory cytokines as well as ROS (such as superoxide ion (O_2_^•−^)) and RNS (including nitric oxide (NO) and peroxynitrite) (19), contributing to inflammatory responses and oxidative/nitrosative stress, exacerbating tissue damage. On the other hand, M2 microglia primarily release protective and trophic factors, thus exerting anti-inflammatory and immunosuppressive effects (20). Activated microglia may also release cytotoxic mediators such as arachidonic acid, glutamate, and histamine (21). NO and O_2_^•−^ are naturally produced during aerobic metabolism and participate in various physiological processes (22, 23). Their overproduction, accompanied by decreased cellular antioxidant defenses, causes the well-known condition of oxidative/nitrosative stress (24) involved in wide number of pathological states, including acute and chronic neurodegenerations (25–27). During oxidative/nitrosative stress, the reaction between the excess levels of NO and O_2_^•−^ generates peroxynitrite, a compound that can damage lipids, proteins, DNA, and mitochondria, leading to inflammation (28) and contributing to neurodegeneration (29).

Amyloid beta (Aβ)1–42 is a peptide physiologically found in the human brain and cerebrospinal fluid (30). The well-recognized hallmarks of AD include extracellular deposition of insoluble Aβ aggregates in the brain and blood vessels (31) as well as the presence of intracellular neurofibrillary tangles composed by hyperphosphorylated tau protein (32). Various factors such as monomer concentration, pH, the presence of metal ions, temperature, and oxidative/nitrosative stress can influence the kinetics of Aβ aggregation (33). Among the different forms of Aβ, oligomers are considered the most toxic, with their toxicity being inversely related to the size of the aggregates (34).

Neuroinflammation is widely recognized as a contributing factor in the development of AD (35). During this process, activated microglia lead to the increased production of pro-inflammatory cytokines such as tumor necrosis factor alpha (TNF-α), interleukin-1 beta (IL-1β), and IL-6 (36). Therefore, activated microglia are implicated in the progression of neurodegenerative disorders (37), and their presence, along with reactive astrocytes, is commonly observed alongside amyloid plaques in the AD brain (38). Macrophages and microglia homeostasis is crucial in the pathophysiology of oxidative stress and inflammation driven disease (39) with the M1/M2 balance representing a new pharmacological target for treating these disorders (40).

Additionally, it has been found that oxidative/nitrosative stress is accompanied by mitochondrial dysfunction (41), causing impairment of energy metabolism with energy crisis (42), with key metabolic enzymes deeply affected by disruption of redox homeostasis (43). Recently, it was found that mitochondrial dysfunction and oxidative/nitrosative stress are associated with changes in the levels of UDP-derivatives (namely, UDP-Galactose = UDP-Gal, UDP-Glucose = UDP-Glc, UDP-N-acetyl-Galactose = UDP-GalNac, UDP-N-acetyl-Glucose = UDP-GlcNac) (44), the role of which in maintaining correct protein glycosylation is of fundamental importance (45). The connection between energy metabolism and ROS is also particularly evident during aging and the progression of age-related diseases like atherosclerosis and neurodegenerative conditions (46, 47).

In the present study, by monitoring the production of NO and ROS along with changes in parameters related to cellular energy metabolism, we investigated the ability of carnosine to mitigate the toxic effects of Aβ1–42 oligomers [used at a concentration known to induce oxidative stress in different *in vitro* models (48–51)] in human microglial cells (HMC3). Novel findings demonstrating that carnosine prevents microglia alteration induced by Aβ1-42, by reducing oxidative stress and counteracting mitochondrial dysfunction, are discussed in light of its potential application in AD.

## 2 Materials and methods

### 2.1 Materials and reagents

C-Chip disposable hemocytometers used for cell counting were supplied by Li StarFish S.r.l. (Naviglio, MI, Italy). HFIP-treated Aβ1–42 was obtained from Bachem Distribution Services GmbH (Weil am Rhein, Germany). Human microglia (HMC3 cells) (ATCC^®^ CRL-3304™) along with Eagle’s Minimum Essential Medium (EMEM), fetal bovine serum (FBS), trypsin-EDTA and penicillin/streptomycin solutions were purchased from American Type Culture Collection (ATCC, Manassas, VA, USA). Anti-Iba1 antibody (011-27991) was obtained from FUJIFILM Wako Pure Chemical Corporation, Richmond, VA, USA). Centrifuge tubes equipped with 3 kDa molecular weight cut-off filters, methanol, water, chloroform, and far-UV acetonitrile were purchased from VWR International (West Chester, PA, USA). Both Sylgard 184 polydimethylsiloxane (PDMS) prepolymer and curing agent, used for the preparation of microfluidic chips were obtained from Ellsworth Adhesives (Germantown, WI, USA). All water used in our study was Ultrapure (18.3 MΩ cm) (Milli-Q Synthesis A10, Millipore, Burlington, MA, USA). The remaining materials, all of analytical grade, were supplied by Sigma-Aldrich Corporate (St. Louis, MO, USA) or Thermo Fisher Scientific Inc. (Pittsburgh, PA, USA) unless specified otherwise.

### 2.2 Preparation of Aβ1–42 oligomers

The oligomers of Aβ1–42 were prepared starting from the monomeric form by using a previously published and validated protocol (52). In summary, lyophilized HFIP-treated Aβ1–42 was dissolved in DMSO at a final concentration of 5 mM. Subsequently, ice-cold DMEM/F12 (1:1) medium was utilized to further dilute the samples to 100 μM. The obtained Aβ1–42 samples were then incubated at 4 °C for 48 hours under gentle rotation. At the end of the incubation, the oligomeric solutions were either used immediately for treating HMC3 cells or aliquoted and stored at −20 °C for future use.

### 2.3 Propagation and maintenance of cells

HMC3 cells were maintained in EMEM medium supplemented with 10% FBS, 0.3 mg/mL streptomycin, 50 IU/mL penicillin, 1 mM GlutaMAX, 1 mM sodium pyruvate, and MEM non-essential amino acids by using 25 cm² or 75 cm² polystyrene flasks within a humidified incubator set at 37 °C with 95% air and 5% CO_2_. Cells were subcultured every 3 to 5 days, depending on the observed confluence.

### 2.4 Analysis of cell viability

HMC3 cells were harvested using a trypsin-EDTA solution, counted with a C-Chip disposable hemocytometer, and seeded into 96-well plates at a density of 2 × 10^4^ cells/well. The next day, the cells were incubated for 24 hours (37 °C, 5% CO_2_) with Aβ1–42 oligomers (2 μM final concentration), at the end of which, cell viability was assessed by using the MTT (3-(4,5-Dimethylthiazol-2-yl)-2,5-Diphenyltetrazolium Bromide) assay (53). Briefly, the medium from each well was removed and the MTT solution (1 mg/mL in EMEM medium) was added to each well, followed by incubation for 2 hours at 37 °C, 5% CO_2_. The MTT solution was then removed and the DMSO was added to melt the formed crystals. As a last step, the absorbance was measured at 569 nm using the Synergy H1 Hybrid Multi-Mode Microplate Reader (Biotek, Shoreline, WA, USA). Resting (untreated) HMC3 cells were used as controls.

### 2.5 Intracellular NO and ROS levels determination

On the day of the experiment, HMC3 cells were harvested by using Trypsin/EDTA, counted, plated in 25 cm^2^ polystyrene culture flasks, and incubated in a humidified environment (37 °C, 5% CO_2_) to allow the complete cell adhesion. The next day, cells were left untreated (control) or treated with Aβ1–42 oligomers (2 µM) in the absence or presence of carnosine (10 mM; 1 hour pre-treatment) for 24 hours. At the end of the treatment, the intracellullar NO levels in HMC3 cells were measured by using a Countess 3 FL Automated Cell Counter with Invitrogen GFP EVOS LED Light Cubes (54), while intracellular ROS levels were quantified through microchip electrophoresis coupled to laser-induced fluorescence (ME-LIF). Cells were labeled with either 4-amino-5-methylamino-2′,7′-difluorofluorescein diacetate (DAF-FMDA) to measure NO intracellular levels (55), or 2′,7′-dichlorodihydrofluorescein dicetate (H2DCFDA) to measure total ROS intracellular levels (9). When performing ME-LIF experiments, 6-CFDA membrane-permeable non-fluorescent dye was used as an internal standard accounting for differences in cell viability, esterase activity, and volume (23). This reagent has been used previously in single cell chemical cytometry experiments using microchip electrophoresis (56, 57).

For NO intracellular levels’ determination, cells were harvested, counted, and centrifuged at 1100 rpm for 5 minutes. At the end of the centrifugation step, the supernatant was removed, and the pellet was resuspended in 200 µL of the labeling solution (Dulbecco’s phosphate buffered saline (DPBS), DAF-FMDA at the final concentration of 100 µM, and probenecid at the final concentration of 2.5 mM), followed by incubation for 30 minutes at 37 °C on a dry bath heating block. Cells were then diluted by adding 800 µL of pre-warmed DPBS (37 °C) and centrifuged again at 1100 rpm for 5 minutes. Before reading the fluorescence by using the automated cell counter, the supernatant was removed, and the pellet was resuspended in DPBS.

For total ROS intracellular levels’ determination, DAF-FMDA was replaced by H2DCFDA. After the dilution (800 µL of pre-warmed DPBS) and centrifugation (1100 rpm for 5 minutes) steps, the supernatant was removed, each cell pellet was resuspended in 50 µL of pure ethanol, and the lysate solution was filtered by using a 3 kDa molecular weight cut-off filter (12000 rpm for 10 minutes). As a final step, 15 µL of each filtered cell lysate was added to 135 µL of running buffer (10 mM boric acid, 7.5 mM SDS at pH 9.2, and 6-CF (fluorescent; internal standard) at the final concentration of 70 nM, to account for the potential variability during electrophoresis runs. During ME-LIF analysis, 20 μL of each solution was used as previously described (51), except for the separations that were performed in reverse polarity mode. Hybrid PDMS-glass microchips with a simple-T geometry were fabricated as described in detail elsewhere (22, 23, 58).

### 2.6 Distribution and correlation analysis

For the cell distribution analysis, the dataset comprised measurements from samples belonging to the three different experimental conditions and included three attributes for each cell: size, circularity, and fluorescent intensity. Before conducting the analyses, data were normalized by using MATLAB R2022b (version: 9.13.0, Natick, Massachusetts: The MathWorks Inc.; 2022) built-in functions “normalize” and “zscore”. The cell dispersion within conditions was calculated as the mean euclidean distance of each cell from the centroid. Unsupervised hierarchical clustering was performed on normalized data using the “clustergram” function in MATLAB R2022b (https://www.mathworks.com/help/bioinfo/ref/clustergram.html) and visualized as a heatmap with overlaid dendrograms to compare overall metabolic profiles and identify condition-dependent patterns (59).

### 2.7 Analysis of metabolites

The analysis of intracellular metabolites in deproteinized samples obtained by HMC3 cells under all our experimental conditions was performed by employing a high-performance liquid chromatography (HPLC) method. Following incubation, cells were pelleted, washed with ice-cold phosphate buffered saline (PBS), and deproteinized. Previously described ion-pairing HPLC methods were employed for the simultaneous separation of various metabolites, including high energy phosphates (ATP, ADP, AMP, GTP, GDP, GMP, UTP, UDP, UMP, CTP, CDP, CMP, and IMP), oxidized and reduced nicotinic coenzymes (NAD^+^, NADH, NADP^+^ and NADPH), glycosylated UDP-derivatives (UDP-Gal, UDP-Glc, UDP-GalNac, and UDP-GlcNac), reduced glutathione (GSH), nitrite and nitrate, and purines and pyrimidines (hypoxanthine, xanthine, uric acid, guanosine, uracil, β-pseudouridine and uridine) (60, 61). The separation was carried out using a Hypersil C-18 column, while the HPLC apparatus consisted of a Spectra SYSTEM P4000 pump system coupled with a highly sensitive UV6000LP diode array detector, equipped with a 5 cm light path flow cell, set up for acquisition between 200 and 400 nm wavelengths (Thermo Fisher Scientific, Rodano, MI, Italy). Each compound in the chromatographic run was identified and quantified by comparing retention times, absorption spectra, and peak areas with those of ultrapure standard mixtures with known concentrations. Different acquisition wavelengths were employed based on the nature of the metabolites analyzed: 260 nm for high-energy phosphates, nicotinic coenzymes, UDP derivatives, purines and pyrimidines, 266 nm for malondialdehyde (MDA) (though levels were not detectable), and 206 nm for GSH, nitrite, and nitrate.

### 2.8 Measurements of phagocytic activity

HMC3 cells were left untreated (control) or treated with Aβ1–42 oligomers (2 µM) and latex beads (amine-modified polystyrene, fluorescent yellow-green; 0.025% v/v), in the absence or presence of carnosine (10 mM; 1 hour pre-treatment), for 6 or 24 hours. After the desired treatment timepoint, cells were immediately fixed in a 4% paraformaldehyde solution. The cells were then stained with primary (anti-Iba1; goat 011-27991) and secondary (donkey anti-goat A-21447) antibodies, and imaged using a Biotek Cytation 5 imaging reader (Santa Clara, CA, USA). Nine images were taken of each well in a spread out 3x3 square grid fashion to get a good representation of each well. Cell counts were obtained using the Biotek Gen 5 Imaging Software. The bead counts were obtained by using a python code. The parameters of the code were optimized such that setting the bead radius to 4 pixels and the threshold value to 75 obtained the best results. After obtaining the cell counts and bead counts, the phagocytic activity in each condition was obtained by calculating the number of beads per cell.

### 2.9 Statistical analysis

Statistical analysis was performed by using Graphpad Prism software (version 8.0) (Graphpad software, San Diego, CA, USA). Student’s *t*-test was employed to assess the statistical differences between two experimental groups, while one-way analysis of variance (ANOVA), followed by Tukey’s *post hoc* test, was used for multiple comparisons. The statistical significance was set at p-values < 0.05. Data are reported as the mean ± SD of at least 3 independent experiments.

## 3 Results

### 3.1 Aβ1–42 oligomers decrease the viability of HMC3 cells

**Figure 1:**
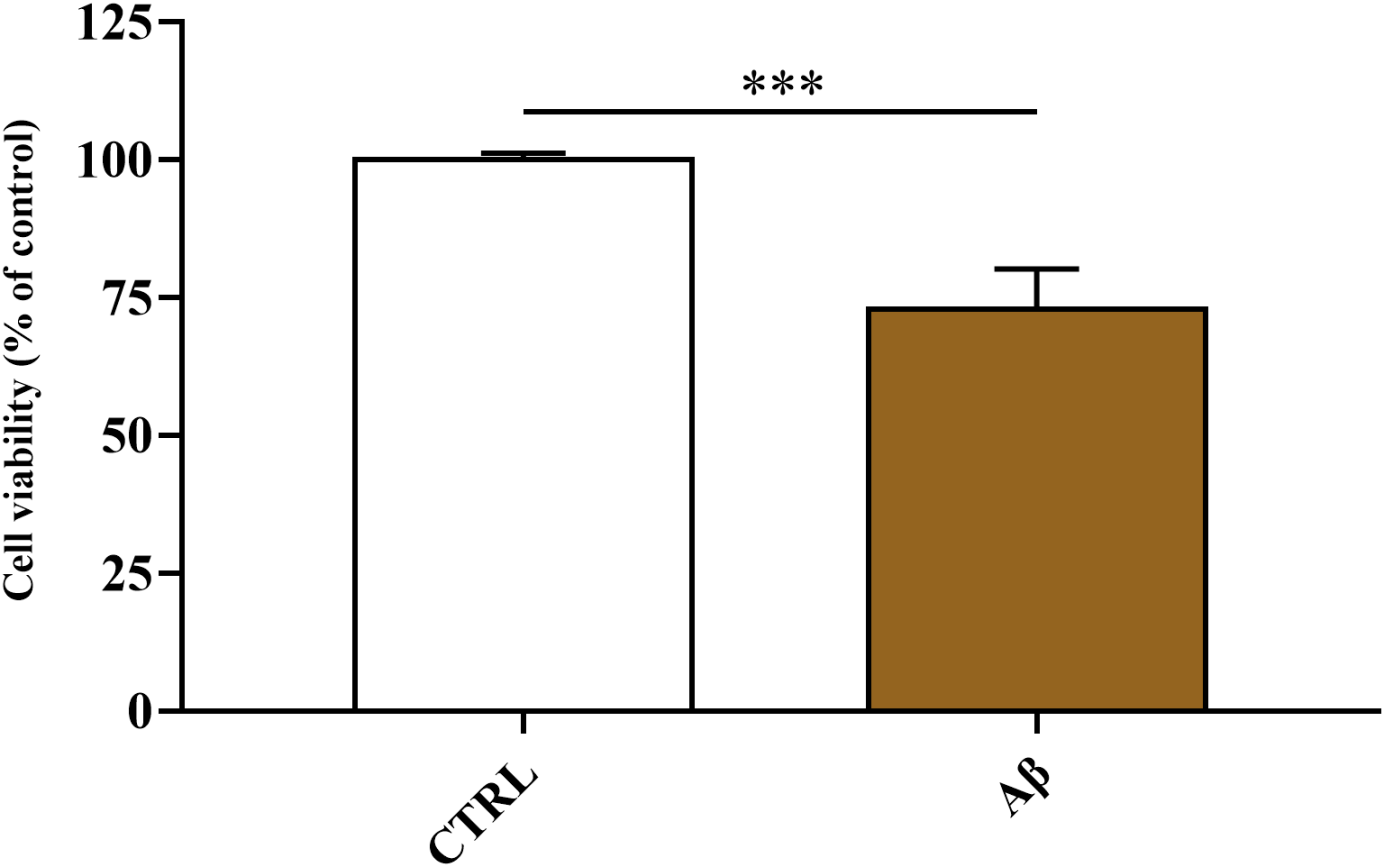
Change in cell viability caused by challenging HMC3 cells with Aβ1–42 oligomers. HMC3 cells were treated for 24 hours with Aβ1–42 oligomers (2 µM). Data are the mean of two independent samples and are expressed as the percent variation with respect to the cell viability recorded in CTRL cells. Standard deviations are represented by vertical bars. Student’s *t*-test was employed to assess the statistical differences between the two experimental groups. ***Significantly different, p < 0.001.

The FIrst aim of the present study was to investigate the changes of metabolic activity and cell viability of HMC3 cells following the exposure to 2 μM Aβ1–42 oligomers, recognized as a concentration capable to induce oxidative stress in different *in vitro* models (48–51). The data reported in **Figure 1** clearly show the detrimental effects induced by the treatment of HMC3 cells with Aβ oligomers for 24 hours, with a significant decreased of cell viability observed in Aβ-treated cells (p < 0.001 compared to controls).

### 3.2 Carnosine counteracts the oxidative stress status induced by Aβ1–42 oligomers in HMC3 cells

**Figure 2:**
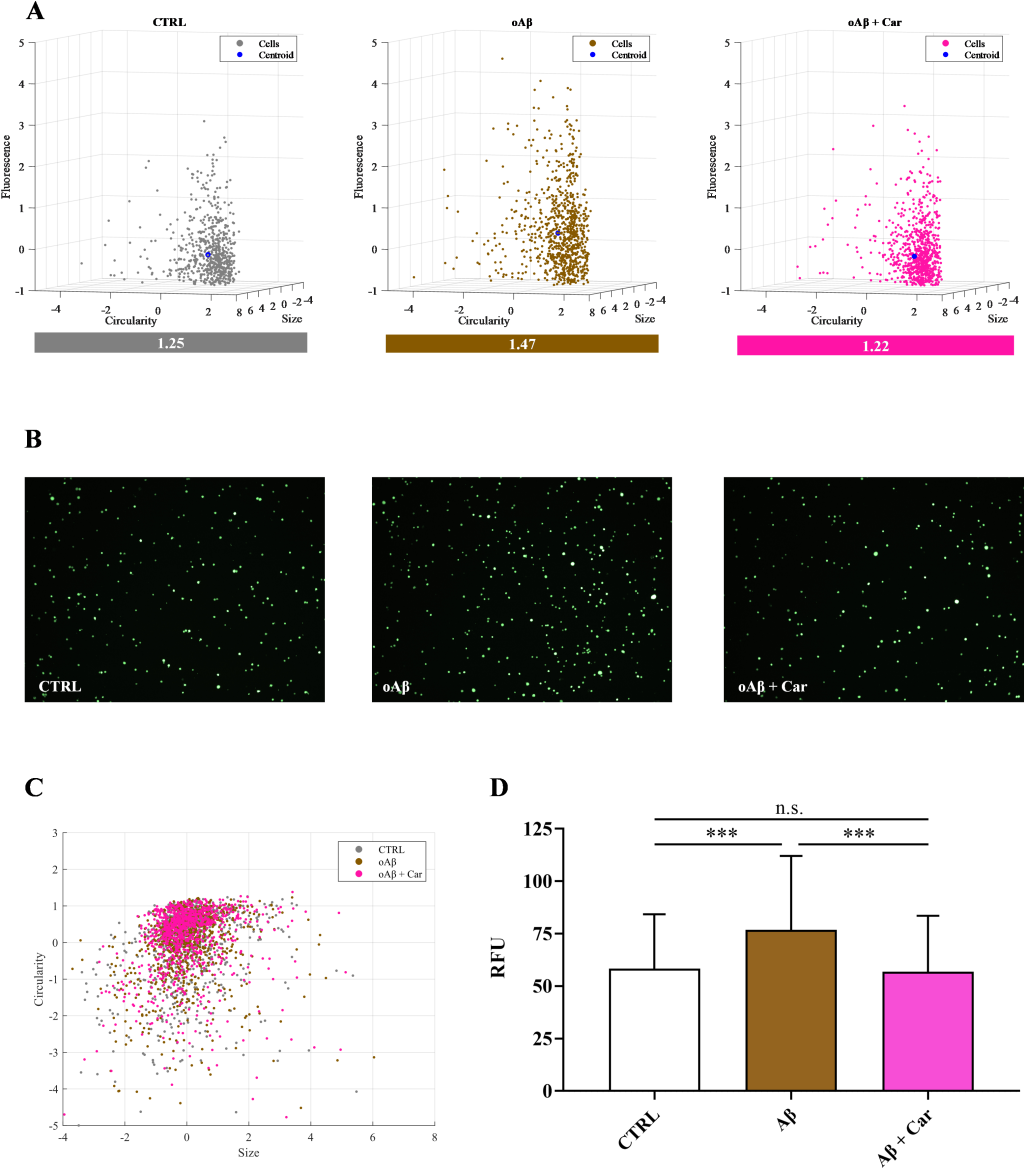
Protective effects of carnosine on the increase of NO intracellular levels induced by Aβ1–42 oligomers in HMC3 cells. **(A)** Cell dispersion analyzed by considering the mean of the distance of each point from the centroid of the distribution. Cytometry data were normalized using the z-score method and are shown in a scatter plot made with MatLab R2022b. **(B)** Representative images of live cells under the indicated treatments obtained by using a Countess 3 FL Automated Cell Counter. **(C)** 2D scatter considering only size and circularity. **(D)** Fluorescence expressed as average Relative Fluorescence Units (RFU) of DAF-FM. Data are means of three independent samples. At least 700 cells per condition were considered. Standard deviations are represented by vertical bars. One-way analysis of variance (ANOVA), followed by Tukey’s *post hoc* test, was used for multiple comparisons. ***Significantly different, p < 0.001.

**Figure 2** illustrates the ability of carnosine to counteract oxidative/nitrosative stress in human microglial cells challenged with Aβ oligomers by decreasing the intracellular levels of NO.

**Figure 2A** clearly depict the heterogeneous response of HMC3 to Aβ1–42 oligomers measured in terms of NO production, as also indicated by mean of the distance of each point from the centroid measured for all the experimental conditions. In particular, a dispersion value of 1.47 was measured in the case of Aβ-treated cells, that was higher than that observed in untreated cells (1.25; -15%). Despite the challenge with Aβ, carnosine was able to restore the basal cellular distribution (1.22), giving fluorescence values significantly lower than those of Aβ (p < 0.001) and almost superimposable to those observed in untreated cells (**Figure 2D**). No notable changes were observed in terms of circularity and size when comparing the different experimental conditions (**Figure 2C**), supporting the notion that the difference in fluorescence among the samples is not due to change in cell morphology, but strictly depends on the different production of NO.

**Figure 3:**
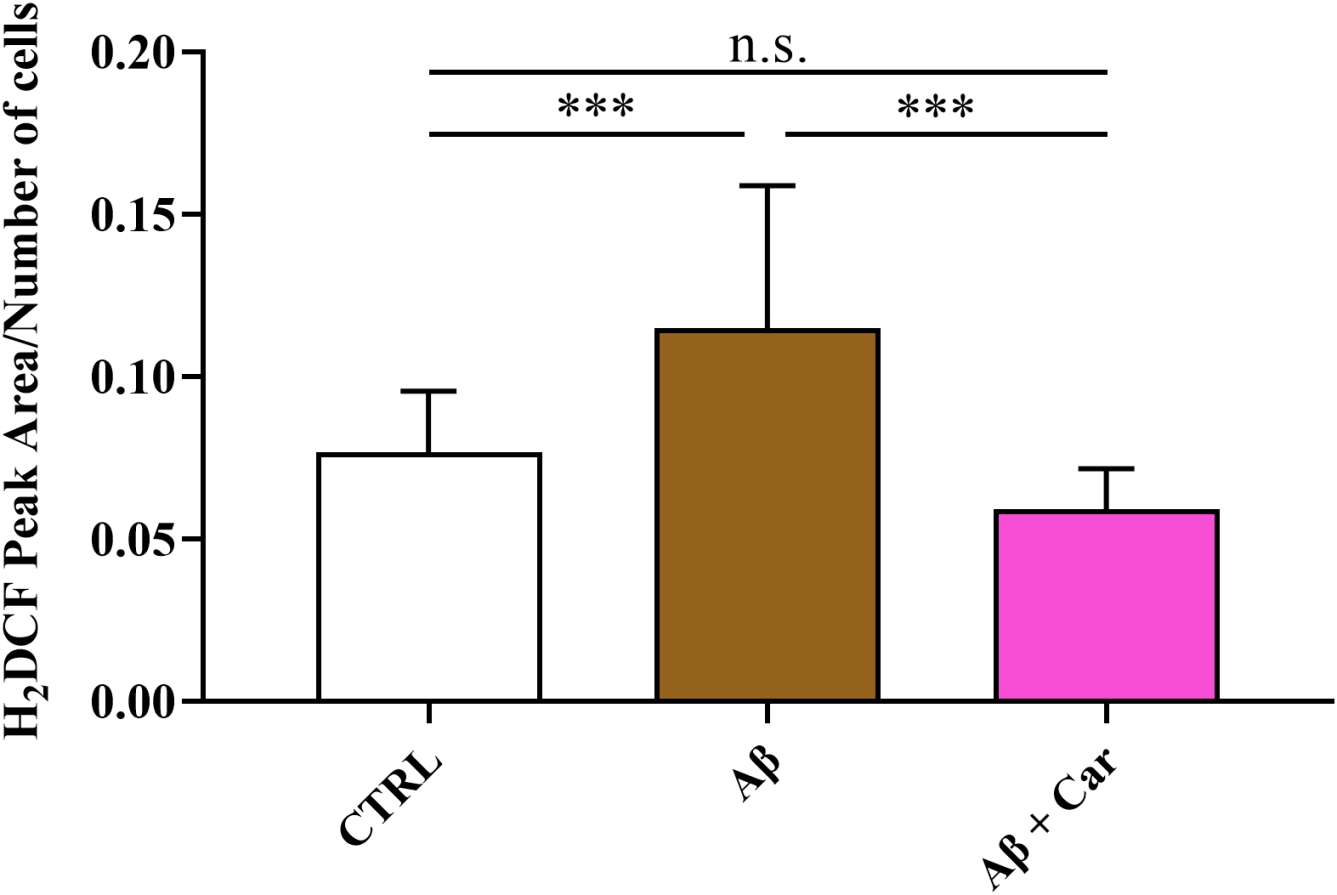
Intracellular concentrations of ROS, expressed as average peak area/number of cells, in resting HMC3 cells and HMC3 cells exposed to Aβ (2 µM, 24 hours), in the absence or presence of carnosine (Car) (10 mM, 1 hour pre-treatment). Data are the mean of three independent samples. Standard deviations are represented by vertical bars. For the normalization of ME-LIF data, the total number of cells and the amplitude of the signal obtained for 6-CF were used. A total of six runs/electropherograms for each sample were considered. One-way analysis of variance (ANOVA), followed by Tukey’s *post hoc* test, was used for multiple comparisons. ***Significantly different, p < 0.001. n.s., not significant.

The ability of carnosine to counteract the increase in NO induced by Aβ in HMC3 cells was corroborated by results of the intracellular levels of ROS, measured under the different experimental conditions. As shown in **Figure 3**, while the treatment of HMC3 with Aβ1–42 oligomers significantly increased the intracellular levels of ROS (p < 0.001 compared to control cells), the presence of carnosine completely restored the basal ROS levels (p < 0.001 compared to Aβ treated cells; not significant compared to control cells).

**Figure 4:**
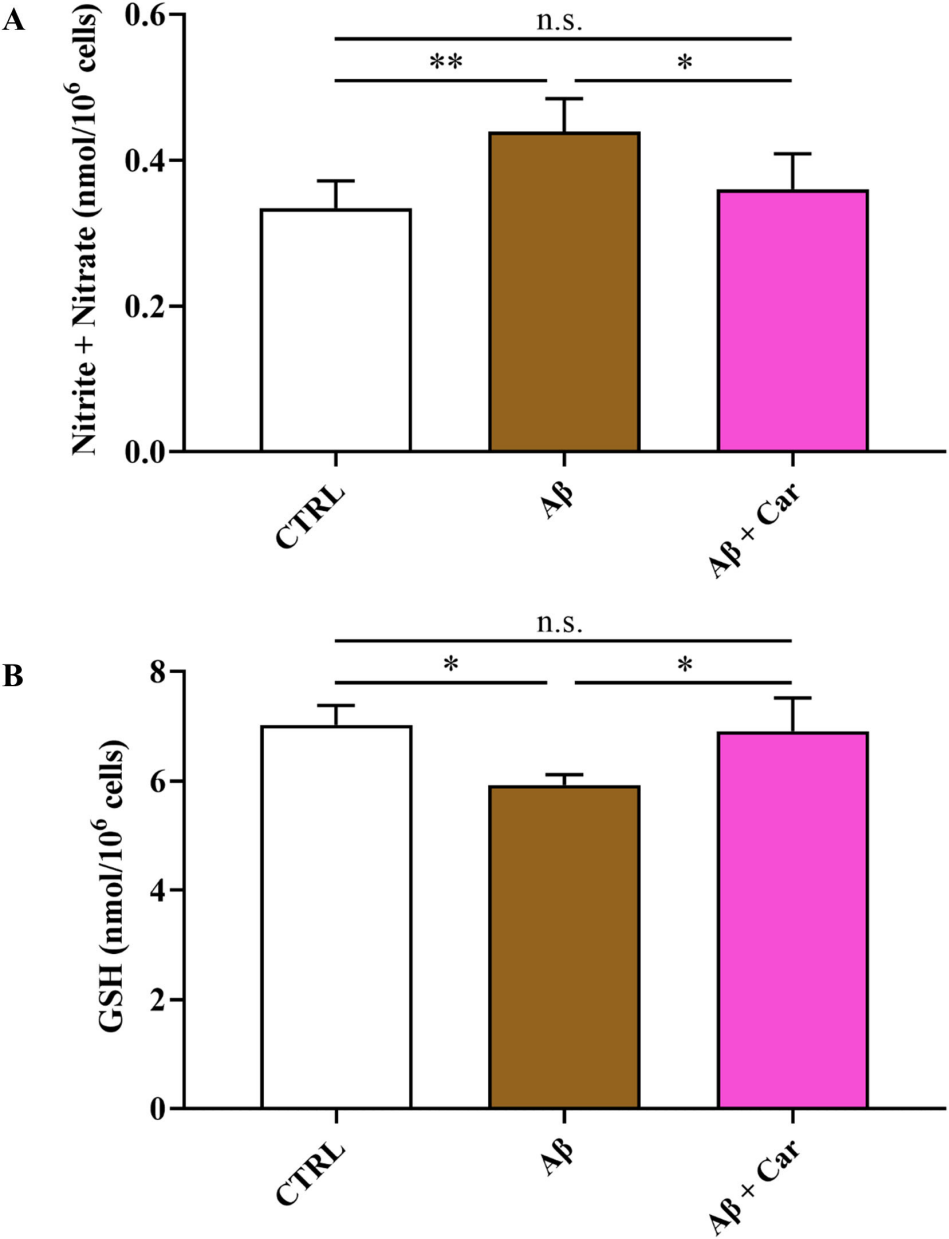
Values of **(A)** nitrite + nitrate (as stable end-products of NO metabolism) and **(B)** GSH determined by HPLC in protein-free extracts of resting HMC3 cells and HMC3 cells exposed to Aβ (2 µM, 24 hours), in the absence or presence of carnosine (Car) (10 mM, 1 hour pre-treatment). Data are the mean of four to five independent samples and are expressed as nmol/10^6^ cells. Standard deviations are represented by vertical bars. One-way analysis of variance (ANOVA), followed by Tukey’s *post hoc* test, was used for multiple comparisons. *Significantly different, p < 0.05; **Significantly different, p < 0.01; n.s., not significant.

Results depicted in **Figure 4** strongly corroborate the previous observations regarding the ability of carnosine to counteract the oxidative/nitrosative stress induced by Aβ.

The exposure of HMC3 to Aβ1–42 oligomers caused sustained formation of the stable end-products of NO catabolism (nitrite + nitrate) (p < 0.01), thereby confirming results obtained by the direct detection of NO performed by ME-LIF (**Figure 2D**). Notably, carnosine significantly inhibited the inductive effects of Aβ on NO formation (p < 0.05 for Nitrite + Nitrate), giving values similar to those observed in control cells. It is also worth mentioning that carnosine was able to rescue the intracellular GSH levels, the principal water-soluble antioxidant molecule, that were significantly lowered by Aβ exposure (p < 0.05).

### 3.3 Carnosine rescues cellular energy metabolism status in HMC3 cells challenged with Aβ1–42 oligomers

**Figure 5:**
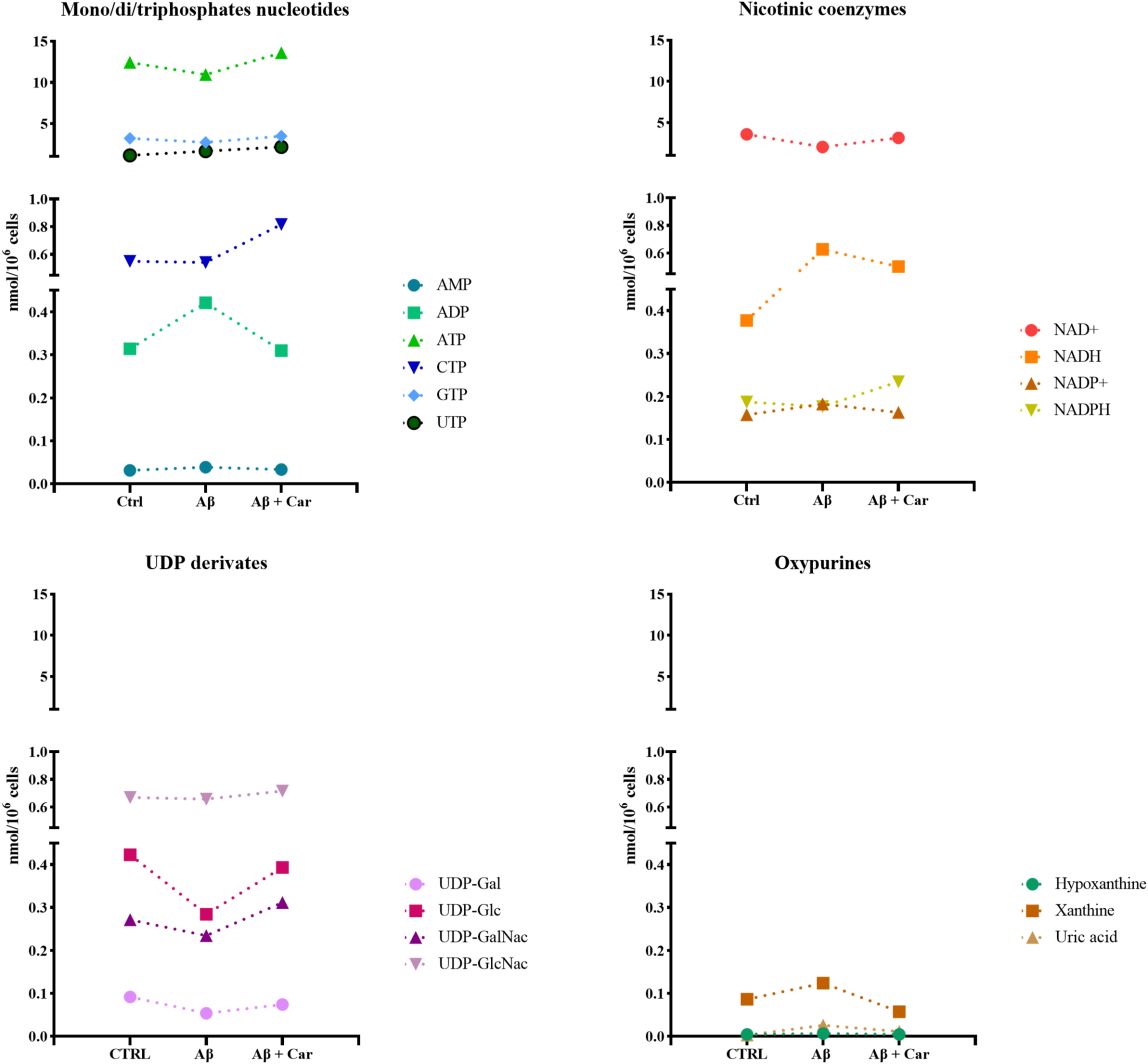
Representation of energy metabolism-related markers under the different experimental conditions, determined by HPLC in protein-free extracts of resting HMC3 cells and HMC3 cells exposed to Aβ (2 µM, 24 hours), in the absence or presence of carnosine (Car) (10 mM, 1 hour pre-treatment). Data show the mean of 5 samples per condition and are expressed as nmol/10^6^ cells.

**Figure 5** gives an overview of the negative effects on parameters reflecting mitochondrial-related energy metabolism and glycosylated UDP-derivatives, when challenging human microglia with Aβ1–42 oligomers.

**Figure 6:**
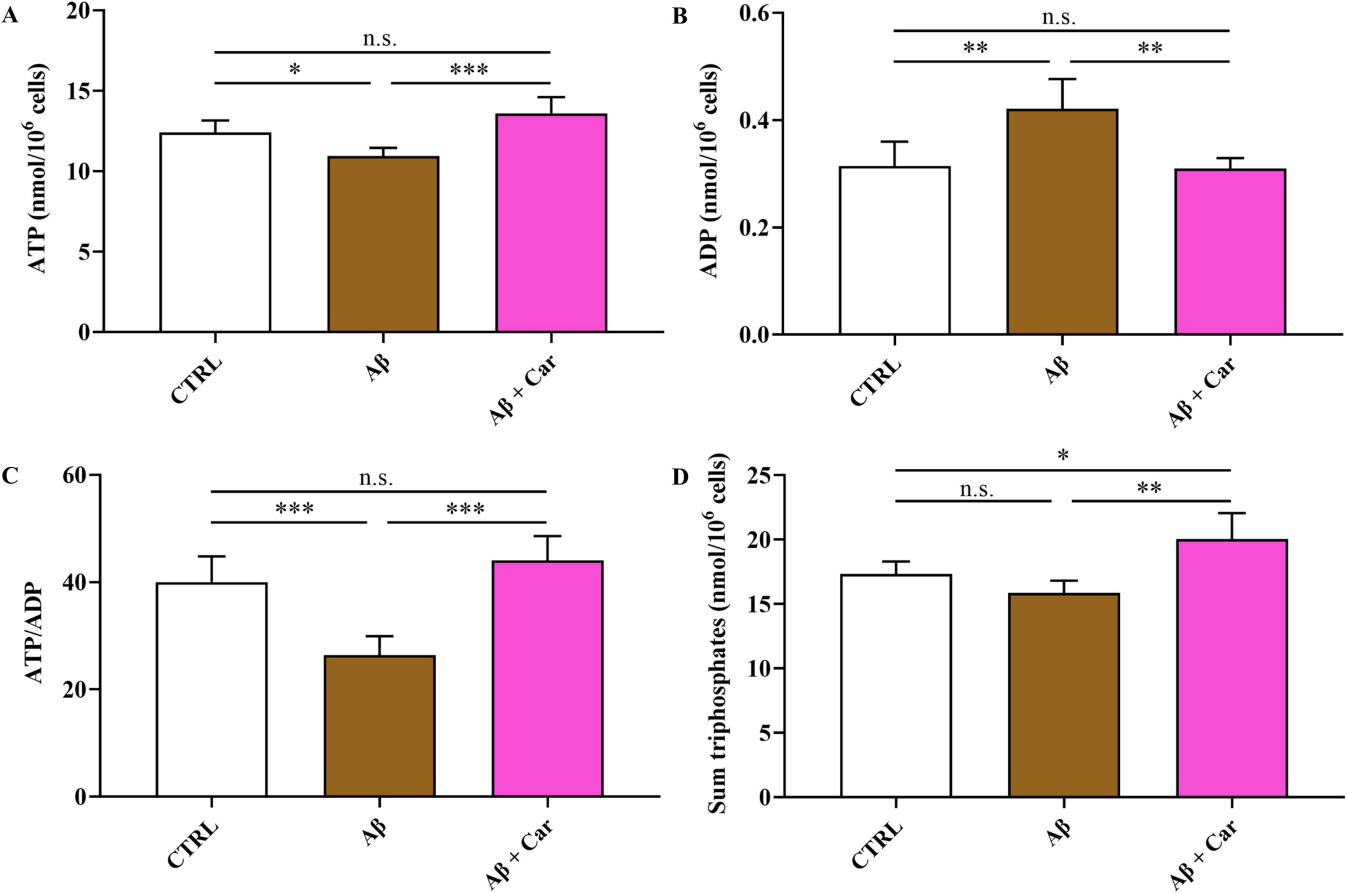
Values of **(A)** ATP, **(B)** ADP, **(C)** ATP/ADP ratio, and **(D)** sum of triphosphates (ATP + GTP + UTP + CTP) determined by HPLC in protein-free extracts in resting HMC3 cells and HMC3 cells exposed to Aβ (2 µM, 24 hours), in the absence or presence of carnosine (Car) (10 mM, 1 hour pre-treatment). Data represent mean of five independent samples and values of intracellular concentrations of ATP, ADP and sum of triphosphates are expressed as nmol/10^6^ cells. Standard deviations are represented by vertical bars. One-way analysis of variance (ANOVA), followed by Tukey’s *post hoc* test, was used for multiple comparisons. *Significantly different, p < 0.05; **Significantly different, p < 0.01; ***Significantly different, p < 0.001. n.s., not significant.

The beneficial effects of carnosine on cell metabolism are illustrated in **Figure 6**, where the concentrations of ATD, ADP, and sum of triphosphate nucleosides (ATP + GTP + UTP + CTP), as well as the values of ATP/ADP ratio, measured in control HMC3 cells and in HMC3 cells challenged with Aβ1–42 oligomers without and with 10 mM carnosine are shown.

The concomitant decline in ATP and increase in ADP, observed in Aβ1-42-treated HMC3 (p < 0.05 and p < 0.01, respectively, compared to the corresponding values of control cells), caused a dramatic reduction of the ATP/ADP ratio from 40.04 (in control cells) to 26.36 (p < 0.001), thus evidencing a decrease of the mitochondrial phosphorylating capacity (62). Under these stressful conditions, the presence of carnosine in HMC3 cells was able to restore all the above-mentioned parameters, allowing cells to maintain correct mitochondrial functions with a better cellular energetic status compared to cells treated with Aβ1–42 oligomers only. Notably, carnosine-treated cells showed energy metabolism parameters similar to untreated control cells (**Figures 6A–C**). The beneficial effects of carnosine on energy metabolism of Aβ-treated cells were corroborated by the higher values of the sum of triphosphate nucleosides compared to both Aβ-treated cells only (p < 0.01), and control cells (p < 0.05).

**Figure 7:**
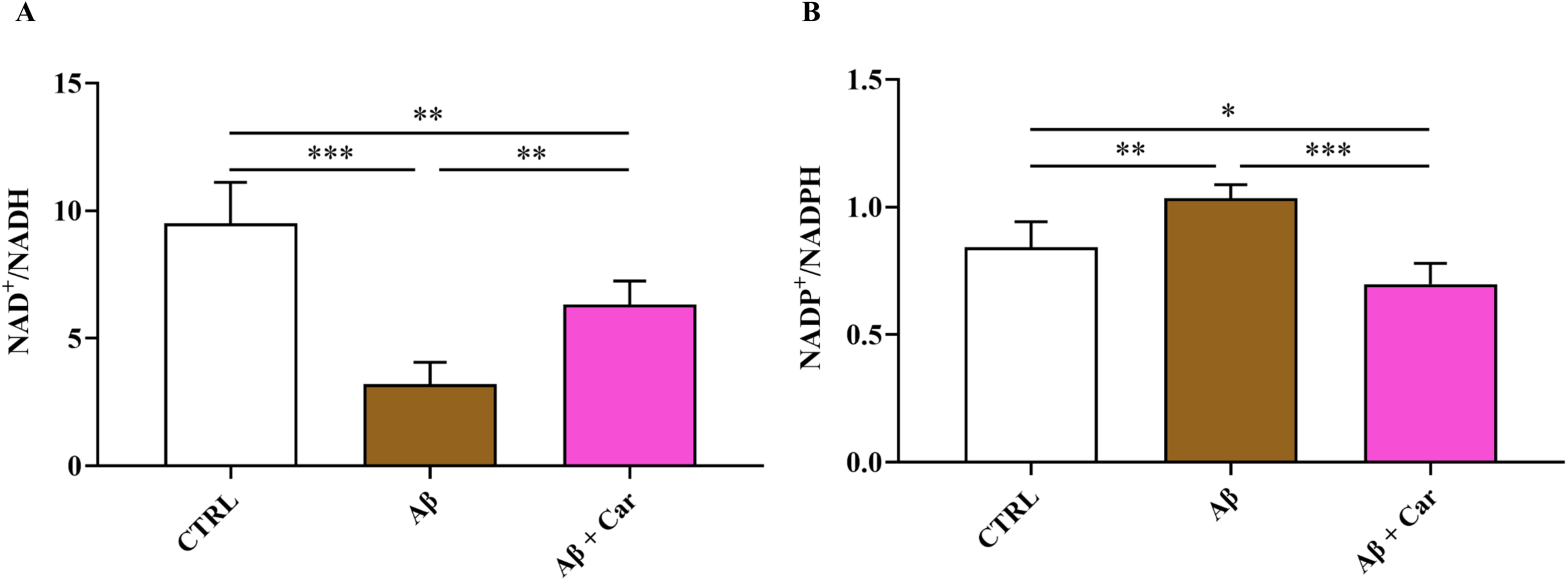
Values of oxidized/reduced ratio of **(A)** NAD^+^/NADH and **(B)** NADP^+^/NADPH determined by HPLC in protein-free extracts in resting HMC3 cells and HMC3 cells exposed to Aβ (2 µM, 24 hours), in the absence or presence of carnosine (Car) (10 mM, 1 hour pre-treatment). Data are the mean of five independent samples. Standard deviations are represented by vertical bars. One-way analysis of variance (ANOVA), followed by Tukey’s *post hoc* test, was used for multiple comparisons. *Significantly different, p < 0.05; **Significantly different, p < 0.01; ***Significantly different, p < 0.001.

The negative impact of Aβ1–42 oligomers on energetic status of human microglia was also testified by the changes of the ratio of oxidized/reduced forms of nicotinic coenzymes. As reported in **Figure 7**, there was a significant decrease in the NAD^+^/NADH ratio (p < 0.001 vs. CTRL) (**Figure 7A**) paralleled by a significant increase in the NADP^+^/NADPH ratio (p < 0.01 vs. CTRL) (**Figure 7B**) as a consequence of Aβ exposure. Change in the NAD^+^/NADH ratio of Aβ1-42-treated cells was due to the concomitant decrease in NAD^+^ concentrations (3.55 ± 0.45 and 2.02 ± 0.55 nmol/10^6^ cells in control and Aβ1-42-treated HMC3, respectively; p < 0.01) and increase in NADH values (0.377 ± 0.038 and 0.628 ± 0.051 nmol/10^6^ cells in control and Aβ1-42-treated HMC3, respectively; p < 0.001), with a net depletion of the NAD^+^ + NADH levels (3.92 ± 0.45 and 2.65 ± 0.57 nmol/10^6^ cells in control and Aβ1-42-treated HMC3, respectively; p < 0.001). Conversely, change of the NADP^+^/NADPH ratio in Aβ1-42-treated HMC3 was simply due to an alteration of the oxidoreductive state of the two forms of the coenzyme, rather than to a net depletion of the NADP^+^ + NADPH sum (0.345 ± 0.019 and 0.359 ± 0.026 nmol/10^6^ cells in control and Aβ1-42-treated HMC3, respectively; n.s.).

Carnosine was able to significantly increase the NAD^+^/NADH ratio (p < 0.001 vs. Aβ), although this value was still lower than that observed in control cells (p < 0.01), and to normalize the NADP^+^/NADPH ratio (p < 0.001 vs. Aβ and n.s. vs. controls). In particular, the increase of the NAD^+^/NADH ratio in Aβ1-42-treated HMC3 with 10 mM carnosine was due to a partial recovery of the NAD^+^ concentration (3.13 ± 0.61 nmol/10^6^ cells; p < 0.05 and n.s. compared to Aβ1-42-treated HMC3 with no 10 mM carnosine and control cells, respectively) and decreased levels of NADH (0.501 ± 0.121 nmol/10^6^ cells; n.s. compared to both Aβ1-42-treated HMC3 with no 10 mM carnosine and control cells). Consequently, the presence of 10 mM carnosine during the challenge with Aβ1–42 allowed HMC3 to have an overall recovery of the NAD^+^ + NADH levels (3.63 ± 0.71 nmol/10^6^ cells; p < 0.05 and n.s. compared to Aβ1-42-treated HMC3 with no 10 mM carnosine and control cells, respectively).

**Figure 8:**
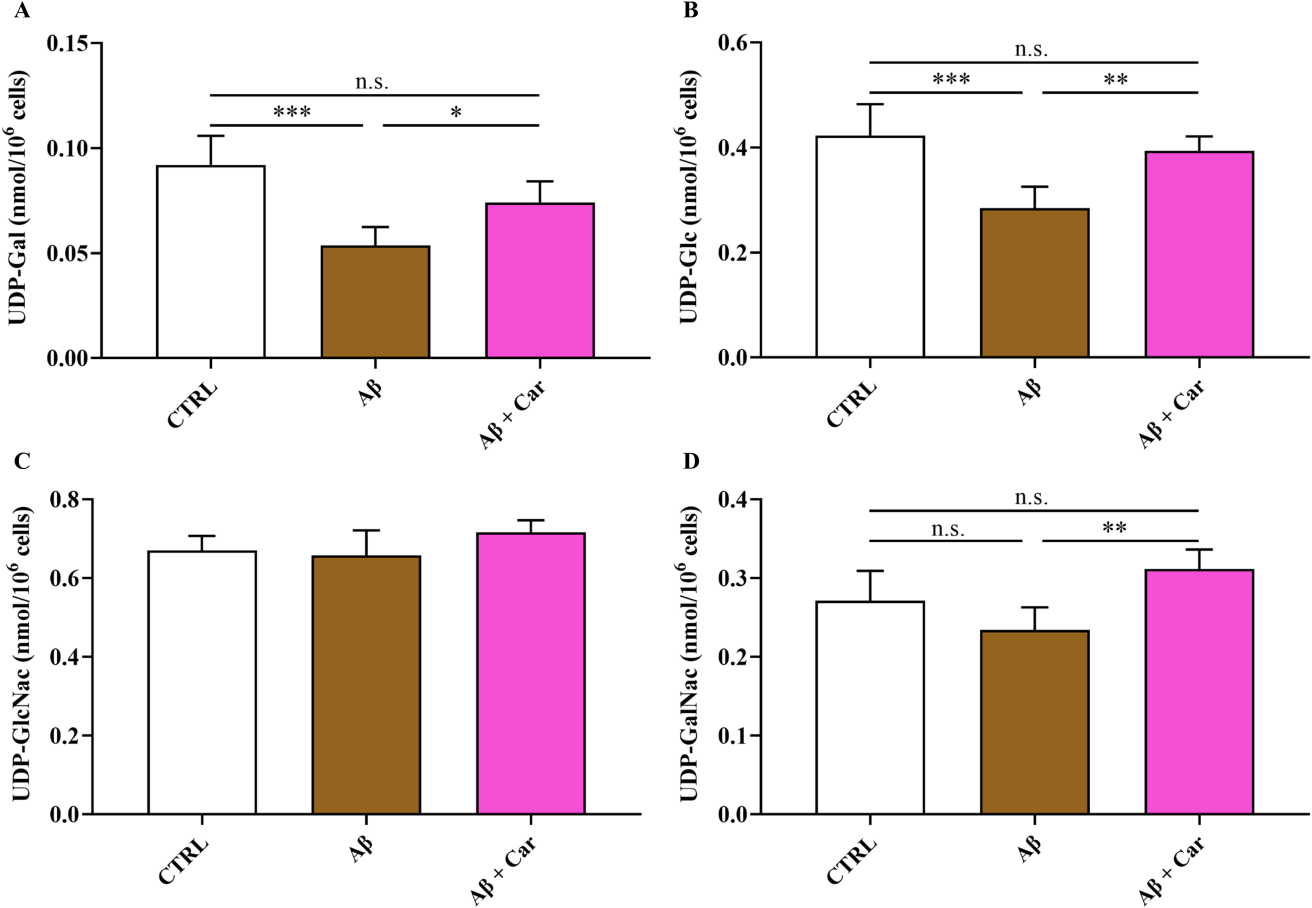
Values of **(A)** UDP-Gal, **(B)** UDP-Glc, **(C)** UDP-GalNac, and **(D)** UDP-GlcNac determined by HPLC in protein-free extracts in resting HMC3 cells and HMC3 cells exposed to Aβ (2 µM, 24 hours), in the absence or presence of carnosine (Car) (10 mM, 1 hour pre-treatment). Data are the mean of five independent samples and are expressed as nmol/10^6^ cells. Standard deviations are represented by vertical bars. One-way analysis of variance (ANOVA), followed by Tukey’s *post hoc* test, was used for multiple comparisons. *Significantly different, p < 0.05; **Significantly different, p < 0.01; ***Significantly different, p < 0.001. UDP-Gal, UDP-galactose; UDP-Glc, UDP-glucose; UDP-GalNac, UDP-N-acetylgalactosamine; UDP-GlcNac, UDP-N-acetylglucosamine. n.s., not significant.

The imbalance of cell metabolism induced by Aβ1–42 also involved the concentrations of the glycosylated UDP-derivatives (UDP-Gal, UDP-Glc, UDP-GalNac, and UDP-GlcNac) (**Figure 8**), ensuring the correct process of protein glycosylation needed for protein trafficking within and outside the cell.

The levels of two out of four (UDP-Gal and UDP-Glc) UDP-derivatives were negatively influenced by the treatment with Aβ oligomers (**Figures 8A, B**). The intracellular levels of each of these compounds were rescued by 10 mM carnosine, highlighting once again, the ability of this dipeptide to counteract the metabolic alterations induced by Aβ oligomers.

**Figure 9:**
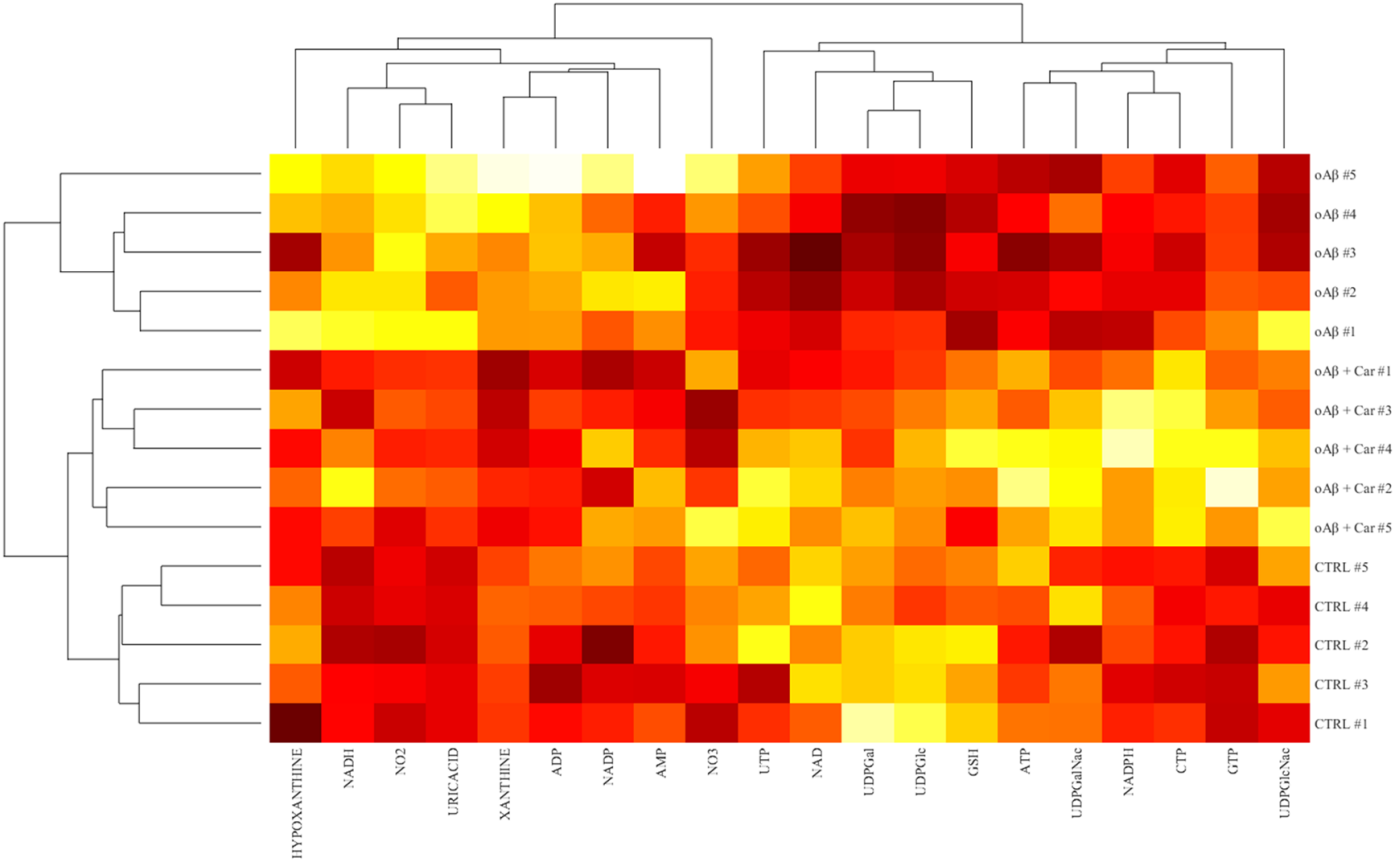
Heatmap of metabolite correlation profiles across the different experimental conditions. The heatmap displays the correlation coefficients of various metabolites (x-axis) in cells treated with the oligomeric form of Aβ (oAβ #1-5), Aβ oligomers in the presence of carnosine (oAβ + Car #1-5), and control (untreated) cells (CTRL #1-5) (y-axis). The color gradient ranges from black (negative correlation) to yellow (positive correlation), with intermediate correlations in shades of red. Dendrograms on the x and y axes illustrate hierarchical clustering, indicating similarities among the metabolites and experimental conditions. Data were analyzed through clustergram function in MatLab R2022b. Distinct clustering patterns are observed between controls and Aβ oligomers, in the presence or absence of carnosine, suggesting differential metabolic correlations in response to the treatments.

**Figure 9** shows the correlation heatmaps with hierarchical clustering. Distinct clustering patterns are evident in the heatmap, with clear separation between the control (untreated) cells, and those treated with the oligomeric form of Aβ, in the presence or absence of carnosine.

The control samples (CTRL #1-5) cluster tightly together, indicating consistent metabolite levels within this group. Moreover, the samples treated with Aβ oligomers (oAβ #1-5) and those treated with Aβ oligomers in the presence of carnosine (oAβ + Car #1-5) form separate clusters, suggesting distinct metabolic response to these treatments. The observed clustering indicates that the treatments induce specific and significant changes in the metabolite expression profiles. The hierarchical clustering method effectively distinguishes between the different groups, underscoring the robustness and reliability of the data. It is also worth underlining that there is a significant correlation between control cells and cells treated with Aβ oligomers in the presence of carnosine, both significantly separated from cell treated with Aβ oligomers only, demonstrating, once again, the overall ability of carnosine to protect microglia metabolism from the toxic effects of Aβ oligomers treatment.

### 3.4 Carnosine significantly enhances the phagocytic activity of HMC3 cells

**Figure 10:**
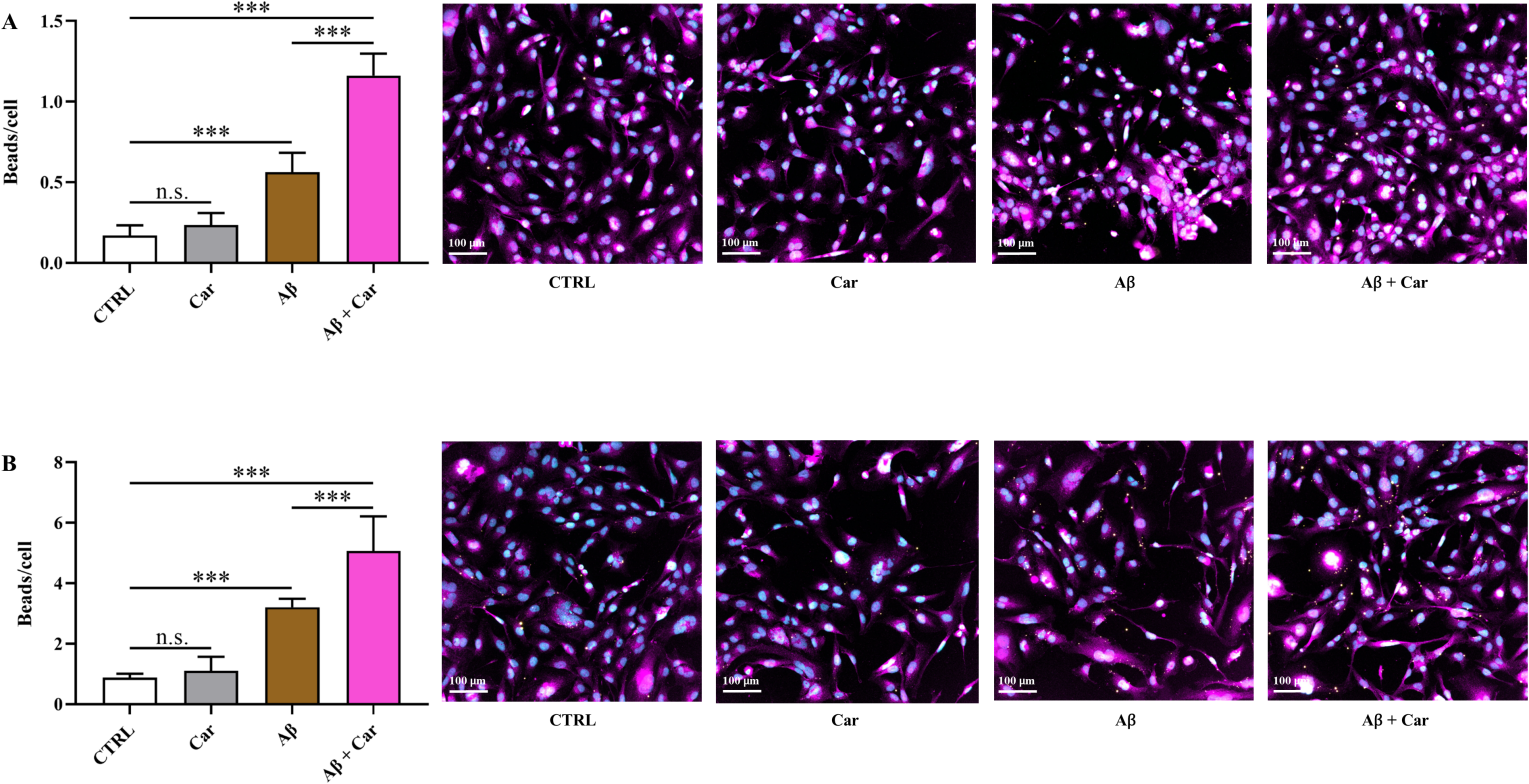
Measurement of microglial phagocytic activity, determined by counting the internalized latex beads amine-modified polystyrene, fluorescent yellow-green, in resting HMC3 cells and in HMC3 cells stimulated with Aβ oligomers (2 µM) at **(A)** 6 or **(B)** 24 hours, in the absence or presence of carnosine (Car) (10 mM, 1 hour pre-treatment). Data are the mean of four to eight independent samples and are expressed as beads/cells. Standard deviations are represented by vertical bars. Scale bar: 100 µm. One-way analysis of variance (ANOVA), followed by Tukey’s *post hoc* test, was used for multiple comparisons. ***Significantly different, p < 0.001.

Given that carnosine has been shown to increase the phagocytic activity of macrophages *in vivo* (63, 64), we wondered whether the antioxidant and energy metabolism-rescuing activities of carnosine was also paralleled by the ability of this dipeptide to increase the phagocytic activity of HMC3 cells. As clearly shown in **Figure 10**, the challenge of HMC3 with Aβ oligomers for both 6 and 24 hours resulted in a significant increase of phagocytosis compared to resting cells (p < 0.001).

Notably, carnosine further increased the phagocytic activity of microglial cells only in the presence of Aβ oligomers, leading to a significantly higher beads uptake compared to resting cells or Aβ-treated cells (p < 0.001). To clarify whether this effect reflected a general stimulatory action of carnosine on phagocytosis, we also treated HMC3 cells with carnosine in the absence of Aβ oligomers, allowing us to prove that the increase in beads uptake was not due to carnosine *per se*; in fact, as clearly depicted in **Figure 10**, no significant differences were observed between resting and carnosine-treated HMC3 cells. These findings showed that carnosine did not act as a basal activator of microglial phagocytosis, but rather enhanced and/or supported the phagocytic response under pathological stress conditions, such as Aβ challenge.

## 4 Discussion

It is well-known that the oligomeric forms of Aβ1–42 peptide represent the most toxic species of Aβ, being able to cause synaptic loss and neuronal death in the brains of individuals with AD (65). Aβ can lead to neuronal death by directly affecting neurons or by inducing the production of inflammatory and toxic factors from microglia or infiltrating mononuclear cells (66). Oxidative/nitrosative stress, caused by an imbalance between pro-oxidants and antioxidants in favor of pro-oxidants and an excess NO production, has a negative impact on cell functions and plays a key role in the pathogenesis of AD (67), occurring earlier than the formation of senile plaques, due to the abnormal deposition of Aβ, and the intracellular accumulation of neurofibrillary tangles, formed because of the hyperphosphorylation of tau protein (68). In the context of Aβ oligomers toxicity, oxidative/nitrosative stress has shown to play a critical role (69). In fact, on one hand, Aβ oligomers are able to impair synaptic plasticity and promote neurodegeneration and neuroinflammation through oxidative/nitrosative stress (70); on the other hand, oxidative/nitrosative stress itself can promote the oligomerization of Aβ peptides, making this peptide even more toxic (71). This bidirectional relationship between Aβ oligomers and oxidative/nitrosative stress underscores the importance of targeting oxidative stress in therapeutic strategies for AD.

Glial cells, and in particular microglia, are involved in the regulation of different physiological processes that include, but are not limited to, the production of trophic factors essential for the processes of proliferation, survival, and differentiation of neurons, as well as to the regulation of synaptic plasticity, learning and memory (18, 72). Microglial dyshomeostasis instead drives to several pathological conditions, including neurodegeneration (73–75). Nowadays, it is well-accepted the dual role played by microglia in the progression of AD; in fact, while during the early stages of AD microglia exert neuroprotective activities, its activation during the late stages of the disease seems to be detrimental (76). Whether microglia have a positive or negative role in AD remains largely controversial and the precise molecular targets for intervention are not well defined.

As previously mentioned, carnosine possesses a multimodal mechanism of action that includes its ability to inhibit the formation of toxic Aβ aggregates (e.g., oligomers) and oxidative/nitrosative stress. Carnosine has also shown a significant regulatory activity toward macrophages and microglia, being able to decrease the expression of pro-oxidant enzymes, thus reducing the production of ROS and RNS, enhance cell antioxidant capacity, improve the formation and release of anti-inflammatory and trophic factors, and boost cellular energy metabolism (8, 58, 77, 78), suggesting that this molecule may be considered as a promising candidate for the treatment of neurodegenerative disorders, including AD. In addition to the above, the treatment of carnosine could also allow the rescuing of the dipeptide physiological levels. In fact, the importance of carnosine homeostasis/levels in humans was demonstrated in a study carried out by Fonteh et al. (79), where a selective deficit of carnosine has been related to cognitive decline in probable AD subjects. According to this scenario, in the present study, we first explored the changes in cell viability induced by Aβ1–42 oligomers on human microglia and then evaluated the correlation between Aβ detrimental effects and the production of NO and total ROS, both significantly contributing to the neurodegenerative phenomena observed in AD (66, 67). When monitoring cell viability under our experimental conditions, we observed that Aβ1–42 oligomers promoted a significant decrease of this parameter (**Figure 1**), that was accompanied by a significant increase in the intracellular levels of both NO and ROS (**Figures 2** and **3**, respectively). Our previous experiments demonstrated that oxidative/nitrosative stress induced by Aβ oligomers in HMC3 cells was paralleled by a depletion of intracellular GSH, the main water-soluble antioxidant (80). Interestingly, our experimental model of Aβ-induced oxidative stress mimicks what observed in patients with AD and mild cognitive impairment, where GSH levels were significantly decreased in the frontal cortex and hippocampus (81, 82). Noteworthy, 10 mM carnosine, the highest non-toxic concentration being able to exert neuroprotection in different *in vitro* models including HMC3 cells, was able to significantly counteract oxidative/nitrosative stress by reducing the intracellular levels of NO (and its related end products) as well as that of total ROS, concomitantly rescuing the intracellular levels of GSH (**Figure 4**). These findings, proving for the first time the ability of carnosine to decrease Aβ-induced oxidative/nitrosative stress in human HMC3 microglial cells, are in line with previously published *in vitro* and *in vivo* results. In these previous studies, carnosine was able to exert multiple effects: it inhibited the production of nitrite, nitrate, and ROS induced by a combination of lipopolysaccharide and ATP in HMC3 cells (83); protected neuronal cells against oxidative/nitrosative stress through the modulation of mitogen-activated protein kinase pathway (84); exerted neuroprotection in primary rat cerebellar cultures stimulated with 2,2’-azobis(2-amidinopropane) dihydrochloride or rotenone, both of them able to increase the amount of intracellular ROS (85); decreased ROS levels in the ischemic brain in a mouse model of permanent focal cerebral ischemia, also preserving basal glutathione levels (64); suppressed the expression of 4-hydroxynonenal, 8-hydroxy-2’ -deoxyguanosine, nitrotyrosine, and receptor for advanced glycation end products in transient middle cerebral artery occlusion (tMCAO) mouse model (86); protected brain microvascular endothelial cells against rotenone-induced oxidative stress *via* histamine H1 and H2 receptors (87). Very recently, the ability of carnosine to mitigate Aβ-induced oxidative stress in mixed glia cells (astrocytes and microglia), by rescuing both ROS and NO intracellular levels, maintaining the *in vivo* architecture, was also demonstrated (54). The mechanism of action involved in the observed antioxidant activity depends on the ability of carnosine to directly scavange RNS, such as NO, and interact with ROS (88), thanks to the well-known proton buffering and metal ion chelating properties of its histidine residue (8). Additionally, carnosine inhibits the Aβ monomer (neuroprotective) to Aβ oligomer (pro-oxidant) transition and disassembles the Aβ aggregates already formed (89, 90). The decrease in intracellular NO levels in microglia cells, may even be related to the inhibition of the expression of inducible nitric oxide synthase (51) and to the increase in the degradation rate of NO into the non-toxic NO end products (8, 91). It is also worth mentioning that an increased loading of carnosine by macrophages (77) and cerebellar (85) cells under stressing conditions has been observed, suggesting an enhancement of the antioxidant power of these cells.

The oxidative/nitrosative stress status induced by Aβ1–42 oligomers in human microglia was coupled to a deep imbalance in the cellular energetics, characterized by mitochondrial dysfunction and a consequent switch toward higher glycolytic rates in order to supply energy production. This was evidenced by the concomitant decrease in the ATP/ADP (**Figure 6**) (indicating decreased mitochondrial phosphorylating capacity) and NAD^+^/NADH (**Figure 7**) (suggesting increased velocity of glycolysis) ratio, typically occurring under various conditions of energy crisis (9, 92, 93). Moreover, the alterations on cell energy metabolism induced by Aβ1–42 oligomers were also demonstrated by the increase in intracellular levels of the sum of oxypurines, thus evidencing an increased rate of ATP consumption with a consequent accumulation of its catabolites.

Our results also evidenced the previously unreported imbalance in the glycosylated UDP-derivatives homeostasis occurring in HMC3 challenged with Aβ1-42 (**Figure 8**). Impacting the hexosamine biosynthetic pathway, through which glycosylated UDP-derivatives are generated (94), have direct impact on the post-translational process of protein glycosylation fundamental for a wide number of cellular processes (95). As previously shown, this phenomenon is with high probability due to endoplasmic reticulum stress occurring under conditions of oxidative/nitrosative stress and mitochondrial dysfunction (44).

The presence of carnosine not only rescued the basal intracellular levels of ATP and ADP, thereby allowing normalization of their ratio, but also led to a significant increase of the total amount of nucleoside triphosphates and a decrease of the sum of oxypurines, suggesting a reinforcement of the cellular metabolic pathways and cycles devoted to the cell energy supply, an ability of carnosine that was already observed in macrophages (RAW 264.7 cells) stimulated with phorbol 12-myristate 13-acetate (58). The presence of carnosine together with toxic concentrations of Aβ oligomers allowed maintenance of correct mitochondrial functions (high values of the ATP/ADP ratio), again switching cell energy metabolism toward oxidative phosphorylation rather than toward glycolysis (increased value of NAD^+^/NADH ratio). In this context it is worth recalling the key role played by the correct nicotinic coenzyme homeostasis in supporting cell energy requirements through electron transfer chain coupled to oxidative phosphorylation, thereby allowing the biosynthesis of structural (e.g., membrane lipids) and functional (e.g., nucleotides) components as well as antioxidant molecules, including GSH (96–98). The previously mentioned decrease in GSH intracellular levels induced by Aβ oligomers may certainly be related to the alterations of NADP^+^/NADPH ratio leading to diminished cell capacity to regenerate GSH under conditions of increased oxidative/nitrosative stress. Our findings are in agreement with previous studies by Li Ouyang et al. (99) and Macedo et al. (100) showing that the treatment with carnosine is able to improve brain bioenergetics under both physiological and stress-induced (e.g., oxygen-glucose deprivation) conditions.

The general amelioration of cell metabolism, induced by the addition of carnosine together with toxic levels of Aβ oligomers, allowed normalization of glycosylated UDP-derivatives. This carnosine effect should allow to restore correct protein glycosylation process, thus ensuring the normalization of protein trafficking (94, 101). These results are in line with a very recent study by Privitera and Cardaci et al. in which the depletion of UDP-derivatives caused by LPS + ATP challenge was counteracted by the treatment with carnosine (83).

By employing a hierarchical clustering method, it was possible to obtain a heatmap of metabolite correlation profiles across the different experimental conditions, allowing to effectively distinguish between the different groups and underlining not only the promising potential of carnosine, but also the robustness and reliability of the data (**Figure 9**). Of note, the significant correlation between controls and cells treated with Aβ oligomers in the presence of carnosine indicates as the presence of the dipeptide is counteracting or, in the best scenario, preventing the dysmetabolism Aβ-induced.

Since carnosine has shown the ability to increase the phagocytic activity of murine macrophages *in vivo* (63, 64), in addition to its antioxidant and free-radical scavenging roles, this possible link was also investigated in human microglia. In a previous study, it has been demonstrated that pre-treatment of RAW 264.7 macrophages with carnosine protected them against Aβ1–42 oligomer–induced toxicity and restored their functional capacity (78). Notably, these protective effects were also associated to a marked improvement in phagocytic activity, measured using antibody-bound tentagel beads, suggesting that carnosine could play a key role in supporting the macrophages’ ability to clear extracellular material under stress conditions. Interestingly, this result was also sustained by the rescue of the chemokine receptor CX3CR1 expression, whose absence has been linked to an impairment of toxic tau internalization by brain macrophages (102). Carnosine also restored and/or enhanced CD11b and CD68 phagocytic markers in murine microglia (103). Accordingly, in the present study, microglia challenged with Aβ1–42 oligomers showed a basal increase in phagocytic activity, which was further significantly improved by the co-presence of carnosine (**Figure 10**). This obtained result confirms the promising role of carnosine in enhancing the phagocytic activity of macrophages/microglia under conditions in which oxidative stress would otherwise impair their immune response.

In summary, the rescue of the cellular energy metabolism status and the remarkable antioxidant activity along with the enhancement of phagocytic activity exerted by carnosine in human microglia challenged with Aβ oligomers might represent a key mechanism contributing to the overall neuroprotective activity of this peptide, but further studies carried out in *in vivo* models of AD are necessary to validate and fully unveil the potential modulatory role of carnosine.

## 5 Limitations of the study and future perspectives

The present study clearly shows the enhanced phagocytic activity as a new feature paralleling the well-known antioxidant activity exerted by carnosine on activate human microglia. Despite that, additional mechanisms should be identified in order to give a better overview of the molecular machinery underlining the promising role of carnosine in the context of AD. In addition to that, the present study was conducted under specific conditions (Aβ oligomers challenge) that cannot fully mimick the complexity of the pathology, and then further translational studies including additional molecular hallmarks (e.g., tau oligomers) are needed to fully comprehend the promising role of carnosine, laying the groundwork for future therapeutic exploration.

## 6 Conclusions

In the present study, we demonstrated for the first time the ability of carnosine to suppress the oxidative/nitrosative stress induced by Aβ1–42 oligomers and to restore cellular energy balance in human microglial cells. In particular, carnosine decreased the intracellular levels of NO and related end products along with total ROS, hypoxanthine, xanthine, and uric acid, also rescuing GSH intracellular levels. The protective activity of carnosine was also mediated by the positive modulation of the mitochondrial phosphorylating capacity (ATP/ADP ratio), the ratio of NAD^+^/NADH and NADP^+^/NADPH nicotinic coenzymes, and UDP-derivatives. The above carnosine modulatory properties were paralleled by increased phagocytic activity. These findings highlight the neuroprotective effects of carnosine on HMC3 cells against Aβ detrimental effects, suggesting its potential as a therapeutic tool in the context of AD and other pathological conditions characterized by microglial overactivation, oxidative stress, and energy imbalance.

## Data Availability

The raw data supporting the conclusions of this article will be made available by the authors, without undue reservation.
